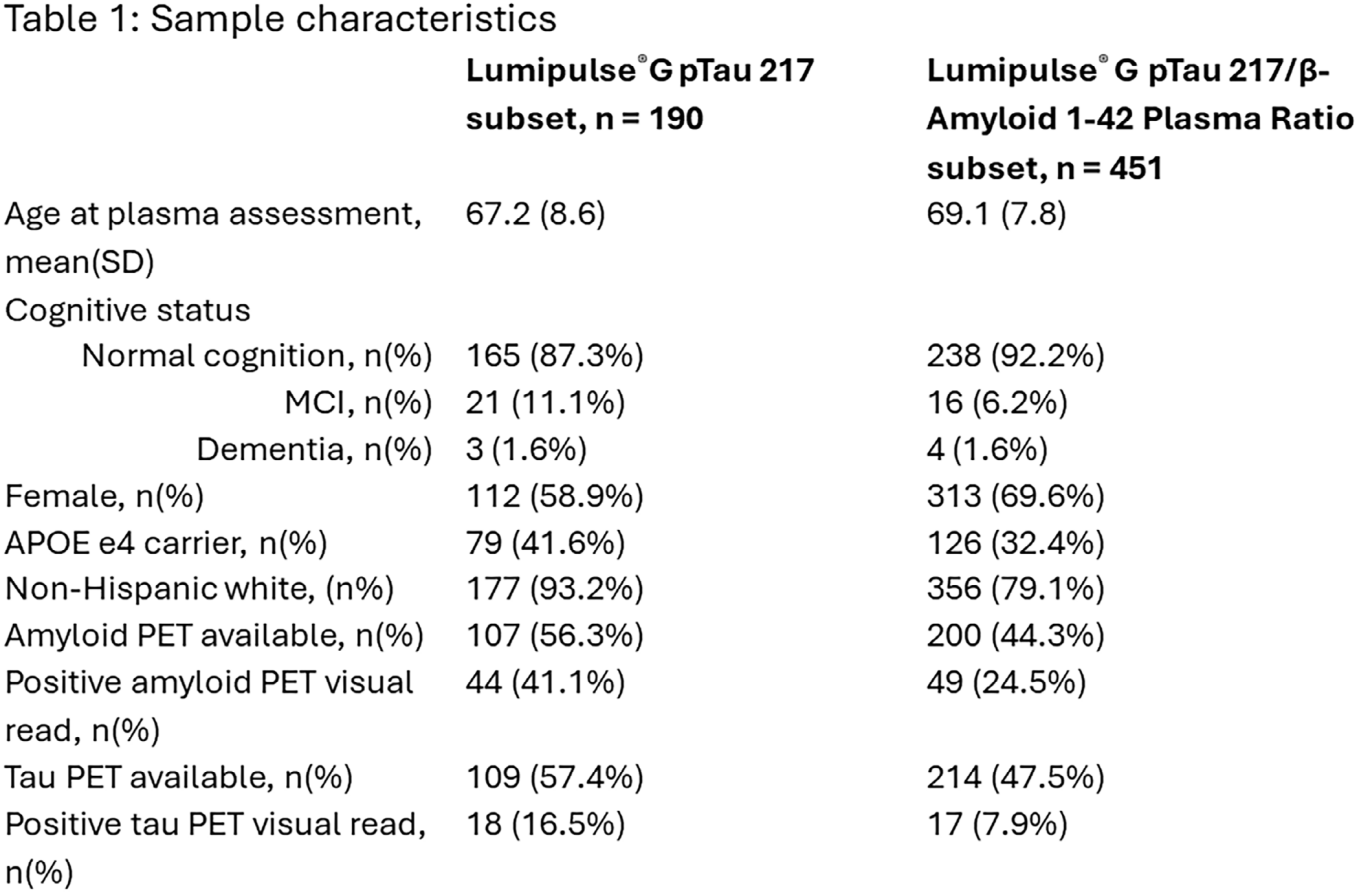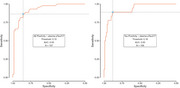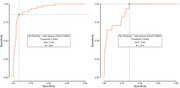# Classification performance of the Lumipulse^®^ G pTau 217 Plasma assay and Lumipulse^®^ G pTau 217/β‐Amyloid 1‐42 Plasma Ratio in a preclinical cohort

**DOI:** 10.1002/alz70856_105672

**Published:** 2026-01-08

**Authors:** Julie E. Oomens, Rachael E. Wilson, Ramiro Eduardo Rea Reyes, Martie Marshall, Nathaniel A. Chin, Sanjay Asthana, Henrik Zetterberg, Sterling C Johnson

**Affiliations:** ^1^ Wisconsin Alzheimer's Disease Research Center, University of Wisconsin School of Medicine and Public Health, Madison, WI, USA; ^2^ Wisconsin Alzheimer's Disease Research Center, University of Wisconsin‐Madison, School of Medicine and Public Health, Madison, WI, USA; ^3^ Wisconsin Alzheimer's Disease Research Center, University of Wisconsin School of Medicine and Public Health, Madison, WI, USA; ^4^ Hong Kong Center for Neurodegenerative Diseases, Hong Kong, Science Park, China; ^5^ Department of Psychiatry and Neurochemistry, Institute of Neuroscience and Physiology, the Sahlgrenska Academy, University of Gothenburg, Molndal, Sweden; ^6^ Department of Neurodegenerative Disease, UCL Institute of Neurology, London, United Kingdom; ^7^ Clinical Neurochemistry Laboratory, Sahlgrenska University Hospital, Gothenburg, Sweden; ^8^ UK Dementia Research Institute at UCL, London, United Kingdom; ^9^ Wisconsin Alzheimer's Institute, University of Wisconsin School of Medicine and Public Health, Madison, WI, USA; ^10^ Wisconsin Alzheimer's Disease Research Center, School of Medicine and Public Health, University of Wisconsin‐Madison, Madison, WI, USA

## Abstract

**Background:**

The Lumipulse^®^G pTau217 assay is among the highest performing plasma assays available for detecting amyloid pathology. Recently, Fujirebio has submitted the Lumipulse^®^ G pTau 217/β‐Amyloid 1‐42 Plasma Ratio to the FDA. The aim of the current study was to assess the utility of these assays for classifying amyloid and tau PET status in a risk‐enriched preclinical Alzheimer's disease cohort.

**Method:**

190 plasma samples from the Wisconsin Registry for Alzheimer's Prevention and the Wisconsin Alzheimer's Disease Research Center were selected and analyzed using the Lumipulse^®^G pTau 217 Plasma assay. Another set of 451 plasma samples were selected and analyzed using the Lumipulse^®^ G pTau 217/β‐Amyloid 1‐42 Plasma Ratio. 38 samples were analyzed using both assays. Amyloid and tau positivity were determined based on visual reads of [C‐11]PiB and [F‐18]Florquinitau PET scans, respectively. We used ROC analyses to assess the amyloid and tau classification performance of the biomarkers. We used Spearman correlations to compare plasma pTau217 levels in the samples that were analyzed using both assays. All analyses were cross‐sectional and the maximum time difference between plasma and PET assessment was two years.

**Result:**

Sample characteristics for both the Lumipulse^®^G pTau 217 and Lumipulse^®^ G pTau 217/β‐Amyloid 1‐42 Plasma Ratio data subsets are provided in Table 1. The ROC AUC of pTau217 was .93 for amyloid PET (95% CI .89‐.98, accuracy 86%) and .93 for tau PET (95% CI .87‐.99, accuracy 88%; Figure 1). The ROC AUC of the pTau217/Abeta 42 ratio was .93 for amyloid PET (95% CI .89 ‐ .97, accuracy 90%) and .90 for tau PET (95% CI .83‐.96, accuracy 68%; Figure 2). The Spearman correlation between plasma pTau217 levels was .92.

**Conclusion:**

Both the Lumipulse^®^G pTau 217 Plasma assay and the Lumipulse^®^ G pTau 217/β‐Amyloid 1‐42 Plasma Ratio accurately classified amyloid and tau status in this largely cognitively unimpaired, preclinical cohort.